# Improved uptake and survival with systemic treatments for metastatic non-small cell lung cancer: younger versus older adults

**DOI:** 10.1186/s12885-023-10800-x

**Published:** 2023-04-19

**Authors:** Bonnie Leung, Aria Shokoohi, Zamzam Al-Hashami, Sara Moore, Alexandra Pender, Selina K. Wong, Ying Wang, Jonn Wu, Cheryl Ho

**Affiliations:** 1 Department of Medical Oncology, BC Cancer – Vancouver Centre, 600 10th Avenue West, Vancouver, BC V5Z 4E6 Canada; 2 Faculty of Medicine and Dentistry, University of Alberta, Edmonton, AB Canada; 3 Department of Medicine, Sultan Qaboos Comprehensive Cancer Care and Research Center, Muscat, Oman; 4 The Ottawa Hospital Cancer Centre, Ottawa Hospital Research Institute, Ottawa, ON Canada; 5 Royal Free London NHS Foundation Trust, London, UK; 6 University of British Columbia Faculty of Medicine, Vancouver, BC Canada; 7 Department of Medical Oncology, BC Cancer, Victoria, BC Canada; 8 Department of Radiation Oncology, BC Cancer, Vancouver, BC Canada

**Keywords:** Lung cancer, Older adults, Systemic therapy, Overall survival, Geriatrics, Cancer Treatment, Aging

## Abstract

**Background:**

Over the past decade, there has been increasing availability of novel therapeutics with improved tolerability and efficacy for advanced non-small cell lung cancer (NSCLC). The study goals were: to compare the uptake of systemic therapy (ST) before and after the availability of targeted tyrosine kinase inhibitors (TKI) and immunotherapy and to examine the changes in overall survival (OS) over time between younger and older adults with advanced NSCLC.

**Methods:**

All patients with advanced NSCLC referred to British Columbia (BC) Cancer in 2009, 2011, 2015 and 2017 were included. One-year time points were based on molecular testing implementation and funded drug availability: baseline (2009), epidermal growth factor receptor TKI (2011), anaplastic lymphoma kinase TKI (2015) and Programed Death-1 (PD-1) inhibitors (2017). Age groups were <70years and ≥70years. Baseline demographics, simplified comorbidity scores (SCS), disease characteristics, and ST details were collected retrospectively. Variables were compared using X2, Fisher’s exact tests and logistic-regression analysis. OS was calculated using the Kaplan-Meier method and compared using the log-rank test.

**Results:**

3325 patients were identified. Baseline characteristics were compared between ages < 70 years and ≥ 70 years for each time cohort with significant differences noted in baseline Eastern Cooperative Oncology Group (ECOG) performance status and SCS. The rate of ST delivery trended upwards over time with age <70 years: 2009 44%, 2011 53%, 2015 50% and 2017 52% and age ≥70 years: 22%, 25%, 28% and 29% respectively. Predictors for decreased use of ST for age <70 years: ECOG ≥2, SCS ≥9, year 2011, and smoking history; and age ≥70 years: ECOG ≥2, years 2011 and 2015, and smoking history. The median OS of patients who received ST improved from 2009 to 2017: age <70 years 9.1 m vs. 15.5 m and age ≥70 years 11.4 m vs. 15.0 m.

**Conclusions:**

There was an increased uptake of ST for both age groups with the introduction of novel therapeutics. Although a smaller proportion of older adults received ST, those who received treatment had comparable OS to their young counterpart. The benefit of ST in both age groups was seen across the different types of treatments. With careful assessment and selection of appropriate candidates, older adults with advanced NSCLC appear to benefit from ST.

## Background

Lung cancer is one of the most commonly diagnosed cancers and the leading cause of cancer-related deaths in Canada and globally [1, 2]. It is predominantly a disease of older persons, with over 70% of all patients with lung cancer being 65 years or older, and nearly 10% are 85 years or older [3]. Despite the prevalence of cancer among older persons, less than 10% of patients are age 75 years or older in phase II and III oncology clinical trials[4]. Older patients are often excluded from registration trials due to multiple chronic comorbidities, polypharmacy, geriatric syndromes, and limited social supports [5, 6]. This under-representation is problematic as it perpetuates uncertainty regarding toxicity and survival outcomes, leading clinicians to extrapolate evidence from younger adults that may not be applicable to the older adult population [5, 7, 8]. Even for the few older adults who are included in clinical trials, they typically have good performance status and no significant comorbidities, and are therefore not representative of most patients seen in real-world practice [6, 9–11].

In the past decade, treatment for advanced NSCLC has changed with increasing availability of novel systemic therapy agents associated with improved tolerability and efficacy, such as tyrosine kinase inhibitors (TKIs) targeting oncogene addicted NSCLC and immune checkpoint inhibitors (ICI) such as anti-programmed death 1 (PD-1) monoclonal antibodies as first-line therapy for advanced NSCLC. The advent of these new treatments has improved survival of the general population of patients with advanced NSCLC [12] but there is insufficient data on whether this is also reflected in the older adult population. Subanalyses of phase 3 clinical trials of TKIs targeting epidermal growth factor receptor (EGFR) mutations [13–15] and anaplastic lymphoma kinase (ALK) rearrangements [16, 17] have consistently shown similar benefits between younger and older adults, but the analyses for older patients are frequently underpowered to demonstrate statistical significance [5]. Similarly, in clinical trials using ICI, older adults derived similar benefits but due to low representation of those aged 75 and over, generalizability of the results for the older population remains limited [5, 7].

Population-based studies can evaluate treatment outcomes outside of the context of randomized controlled trials and examine the comparability of findings in the real-world setting. We conducted a retrospective cohort study to examine how treatment of patients with NSCLC have evolved over time with the arrival of new systemic therapy agents in real-world practice with a particular focus on the older adult population. The objectives of this study were to compare the differences between younger and older adults with advanced, stage IV NSCLC in the uptake of systemic therapy before and after the availability of targeted therapy and ICI, and the changes in median overall survival (OS) over time.

## Methods

This was a retrospective cohort study of patients with stage IV NSCLC seen at British Columbia (BC) Cancer, a publicly funded, provincial comprehensive cancer care program in BC, Canada. There are six regional centres across the province and over 50 community oncology network sites that serve a population of 5.2 million residents [18]. Reports to the Canadian Cancer Registry and the BC Cancer Surveillance and Outcomes Unit show approximately 80% of patients with advanced NSCLC are referred to the provincial program. BC Cancer is a single-payer healthcare system and as such, has complete records on the billing and prescribing of all cancer therapies in the province. Eligibility and exclusion criteria for treatment funding decisions are defined by each tumour group’s content experts and approved by the provincial systemic therapy program. All approved therapies are fully covered by the provincial healthcare program. All patients with stage IV NSCLC who were referred and seen at BC Cancer were included in the analysis.

### Time cohorts

Four one-year time cohorts (January to December of each year) were created based on the implementation of molecular testing and availability of provincially funded drugs for patients with advanced NSCLC. The 2009 cohort was the baseline as there was no funded access to molecular testing nor provincially funded treatments associated with molecular aberrations or immunotherapy. The 2011 cohort captured changes after the implementation of EGFR testing and provincial funding of first-line gefitinib for patients with EGFR mutation positive NSCLC (started in October 2010). The 2015 cohort captured changes after the implementation of ALK testing and provincial funding of crizotinib, which was first approved as second line treatment in March 2014 and as first line treatment in December 2015. Finally, the 2017 cohort captured changes after implementation of programmed death-ligand 1 (PD-L1) testing and provincial funding of ICI (second line with nivolumab in March 2017 and first line with pembrolizumab in February 2018).

### Data collection

Data was collected through the Outcomes and Surveillance Integrated System database and electronic medical records. Cancer staging was based on the American Joint Committee on Cancer staging for NSCLC. Version 6 was used for the 2009 cohort and version 7 was used in all subsequent time cohorts. Clinical information was collected retrospectively. Baseline characteristics, including age, sex, smoking history, Eastern Group Cooperative Group (ECOG) performance status (PS), and histology were obtained from the BC Cancer Surveillance and Outcomes Unit that collects data for the Canadian Cancer Registry. Missing data was manually extracted from patient records. Age groups were categorized as younger (<70 years of age) and older (≥70 years of age). Information regarding death date was provided through linkage with Canadian Vital Statistics.

Systemic therapy details were collected retrospectively through the provincial pharmacy database if patients received any systemic therapy between date of diagnosis and date of death or last follow up. Information collected includes drug names, type of treatment, which line of therapy, and the number of lines of therapy administered. Patients were recorded as having received immunotherapy if they received treatment containing ICI, including combinations with chemotherapy but excluding patients with driver mutations treated with targeted therapy.

Comorbid conditions were manually collected from patient medical records to calculate the simplified comorbidity score (SCS). The SCS was developed in 2005 by Colinet et al. as a simplified and alternative prognostic tool specifically designed for patients with NSCLC. In comparison to the Charlson Comorbidity Index, it has statistical concordance with a κ coefficient of reliability of 0.288 (p < 0.00001). A SCS score of greater than 9 is associated with poor outcome [19]. The weighted comorbidities include tobacco consumption, diabetes, renal insufficiency, respiratory comorbidity, cardiovascular comorbidity, neoplastic comorbidity, and alcoholism.

### Statistical analysis

Descriptive statistical analyses were utilized; frequency of occurrences and percentages were calculated for each of the independent variables. Univariate analysis using chi-squared tests and Fisher’s exact tests were performed to compare age groups based on sex, histology, smoking status, ECOG PS, SCS, and treatment type. Multivariate analysis was conducted using logistic-regression analysis. Statistical significance was defined using two-tailed tests with *p*-value threshold of < 0.05. OS was calculated from the date of diagnosis of stage IV NSCLC to date of death for patients who received best supportive care and from initiation of systemic therapy to date of death for those who received treatment. OS was estimated using the Kaplan-Meier method and compared using the log-rank test. Multivariate analysis of OS was performed using the Cox regression model. Patients for whom data on survival status were missing or were known to be alive at the time of survival data retrieval were censored for the OS outcome. All statistical analyses were conducted using IBM SPSS Statistics software, version 26 (IBM Corp).

## Results

### Demographic and clinical characteristics

A total of 3325 patients with stage IV NSCLC were referred and seen at BC Cancer during the study period: 580 in 2009, 778 in 2011, 1001 in 2015, and 966 in 2017. The median duration of follow up was 5.1 months. The data cut-off date was May 1, 2021. The median age of the entire cohort was 69 years, 61 years for the <70 years cohort and 77 years for the ≥70 years cohort. Of the 1634 adults aged ≥70, 36.1% were aged ≥80 years (n = 591). The distribution of sex, histology, mutation status, ECOG PS, SCS, and smoking status by year is summarized in Table 1. In 2011, 2015 and 2017 there was a statistically significant difference in the distribution of ECOG PS between the <70 and ≥70 age groups. Across all years the proportion of patients with SCS ≥9 was higher in the in older adult group.

**Table 1:** Baseline Characteristics of patients with advanced NSCLC

	2009 (n = 580)			2011 (n = 778)			2015 (n = 1001)			2017 (n = 966)		
	Age <70 (n = 311)	Age ≥70 (n = 269)	p-value	Age <70 (n = 417)	Age ≥70 (n = 361)	p-value	Age <70 (n = 485)	Age ≥70 (n = 516)	p-value	Age <70 (n = 478)	Age ≥70 (n = 488)	p-value
**Median Age at diagnosis (IQR)**	61 (55–65)	76 (73–81)	< 0.001	62 (56–66)	76 (73–81)	< 0.001	61 (57–66)	77 (73–82)	< 0.001	62 (57–65)	78 (73–83)	< 0.001
**Sex** Female Male	147 (47%) 164 (53%)	130 (48%) 139 (52%)	0.803	208 (50%) 209 (50%)	173 (48%) 188 (52%)	0.615	262 (54%) 223 (46%)	258 (50%) 258 (50%)	0.206	249 (52%) 229 (48%)	225 (46%) 263 (54%)	0.071
**Histology** Non-squamous Squamous NOS	129 (42%) 33 (11%) 149 (48%)	94 (35%) 52 (19%) 123 (46%)	0.010	280 (67%) 58 (14%) 79 (19%)	215 (59%) 64 (18%) 82 (23%)	0.087	328 (68%) 71 (14%) 86 (18%)	308 (60%) 83 (16%) 125 (24%)	0.020	312 (65%) 58 (12%) 108 (23%)	307 (63%) 61 (12%) 120 (25%)	0.725
**EGFR Status** Positive	3 (< 1%)	0		32 (8%)	22 (6%)	0.387	50 (10%)	40 (8%)	0.157	72 (15%)	49 (10%)	0.018
**ALK Status** Positive	0	0		1 (< 1%)	0		15 (3%)	3 (< 1%)	0.003	15 (3%)	1 (< 1%)	< 0.001
**ECOG** 0–1 ≥2 Unknown	116 (37%) 171 (55%) 24 (8%)	78 (29%) 166 (62%) 25 (9%)	0.104	178 (43%) 176 (42%) 63 (15%)	105 (29%) 203 (56%) 53 (15%)	< 0.001	182 (37%) 240 (50%) 63 (13%)	133 (26%) 316 (61%) 67 (13%)	< 0.001	169 (35%) 258 (54%) 51 (11%)	118 (24%) 313 (64%) 57 (12%)	0.001
**SCS** <9 ≥9	240 (77%) 71 (23%)	115 (43%) 154 (57%)	< 0.001	295 (71%) 122 (29%)	167 (46%) 194 (54%)	0.001	333 (69%) 151 (31%)	207 (40%) 309 (60%)	< 0.001	330 (69%) 148 (31%)	195 (40%) 293 (60%)	< 0.001
**Smoking** Never Ever Unknown	41 (13%) 268 (86%) 2 (1%)	27 (10%) 240 (89%) 2 (1%)	0.499	57 (13%) 358 (86%) 2 (1%)	48 (13%) 298 (83%) 15 (4%)	0.002	81 (17%) 391 (80%) 13 (3%)	88 (17%) 408 (79%) 20 (4%)	0.555	83 (17%) 389 (82%) 6 (1%)	69 (14%) 404 (83%) 15 (3%)	0.070
**Lines of therapy** Best Supportive Care 1 2 ≥3	173 (56%) 58 (18%) 43 (14%) 37 (12%)	208 (77%) 29 (11%) 16 (6%) 16 (6%)	< 0.001	196 (47%) 78 (19%) 73 (17%) 70 (17%)	271 (75%) 44 (12%) 30 (8%) 16 (5%)	< 0.001	240 (50%) 150 (31%) 56 (11%) 39 (8%)	372 (72%) 98 (19%) 29 (5%) 19 (4%)	< 0.001	229 (48%) 118 (25%) 81 (17%) 50 (10%)	342 (70%) 91 (19%) 38 (8%) 17 (3%)	< 0.001

### Uptake of treatments

**Fig. 1:**
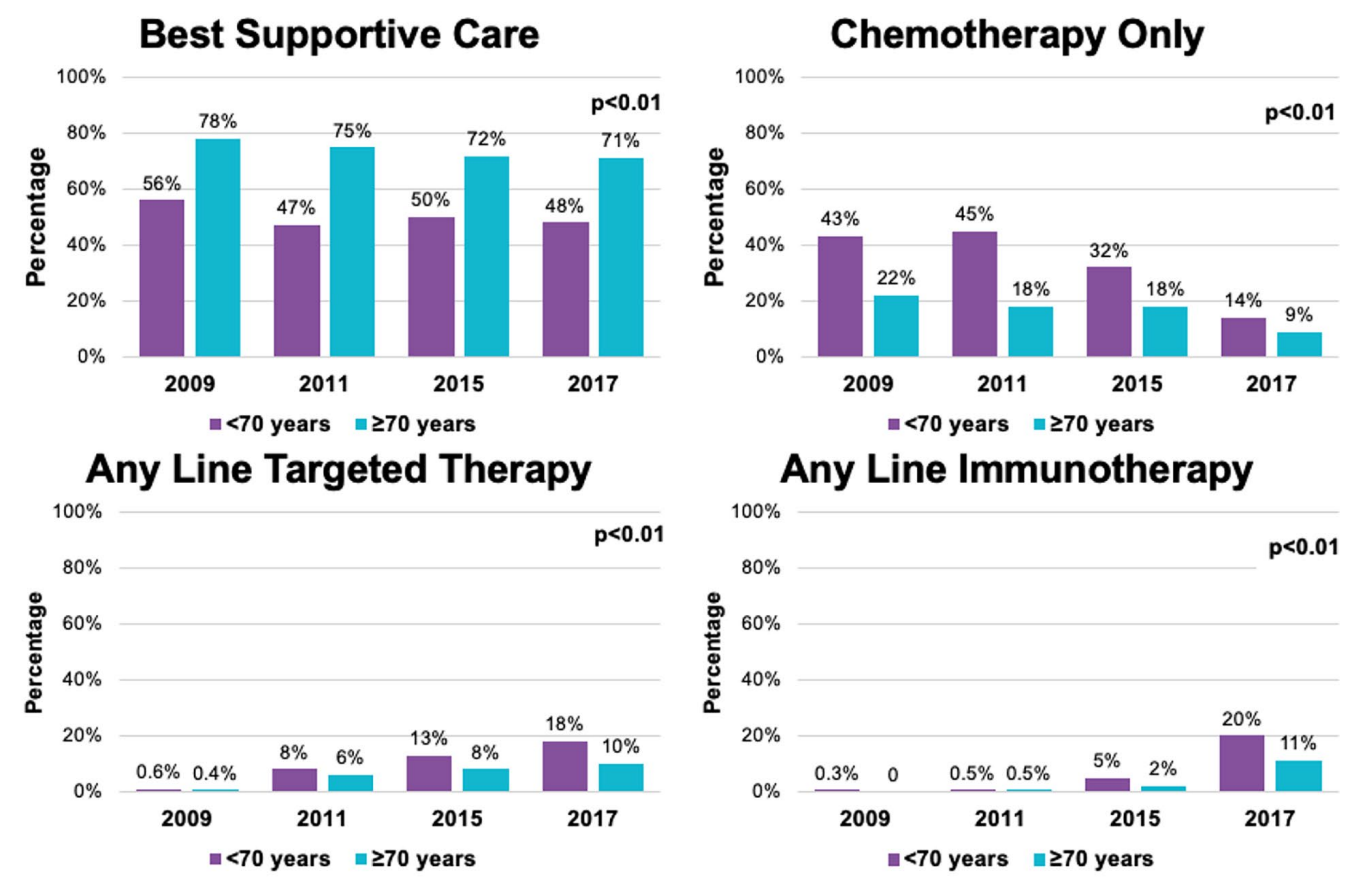
Comparing the uptake of systemic therapy between < 70 and ≥70 over time

Uptake of systemic therapy for adults age <70 years increased from 2009 (44%) to 2011 (53%), and remained stable in the later time cohorts, while the uptake of systemic treatment consistently increased across the four one-year time cohorts for older adults age ≥70 years from 23 to 25%, 28%, and 30% respectively (Fig. 1). The proportion of patients who received second and third line therapy had initially increased in 2011 but decreased thereafter for both age groups (Table 2).

**Table 2:** Systemic therapy details of patients with advanced non-small cell lung cancer by year and age cohort

	2009			2011			2015			2017		
	< 70 years (n = 311)	≥70 years (n = 269)	*p*-value	< 70 years (n = 417)	≥70 years (n = 361)	*p*-value	< 70 years (n = 485)	≥70 years (n = 516)	*p*-value	< 70 years (n = 478)	≥70 years (n = 488)	*p*-value
**First line treatment**	138 (44%)	61 (23%)	0.258	221 (53%)	90 (25%)	< 0.001	245 (51%)	144 (28%)	0.002	249 (52%)	146 (30%)	0.032
% of first line treatments received Platinum doublet Single agent Targeted therapy Immunotherapy Other	121 (88%) 15 (11%) 2 (1%) 0% 0%	50 (82%) 11 (18%) 0% 0% 0%		187 (85%) 8 (3%) 26 (12%) 0% 0%	58 (65%) 12 (13%) 20 (22%) 0% 0%		182 (74%) 4 (1%) 58 (24%) 1 (1%) 0%	87 (60%) 12 (8%) 43 (30%) 2 (2%) 0%		143 (57%) 1 (1%) 82 (33%) 20 (8%) 3 (1%)	71 (48%) 4 (3%) 47 (32%) 23 (16%) 1 (1%)	
**Second line treatment**	82 (26%)	32 (12%)	0.408	143 (34%)	47 (13%)	0.833	96 (20%)	48 (9%)	0.797	131 (27%)	55 (11%)	0.146
% of second line treatments received Platinum doublet Single agent Targeted therapy Immunotherapy Other	9 (11%) 27 (32.9%) 46 (56.1%) 0% 0%	1 (3%) 11 (34%) 20 (63%) 0% 0%		23 (16%) 42 (29%) 75 (52%) 2 (1%) 1 (1%)	7 (15%) 13 (28%) 26 (55%) 0% 1 (2%)		15 (16%) 31 (32%) 32 (33%) 16 (17%) 2 (2%)	6 (13%) 16 (33%) 19 40%) 7 (14%) 0%		22 (17%) 5 (4%) 32 (24%) 69 (53%) 2 (2%)	3 (6%) 1 (2%) 18 (33%) 22 (60%) 0%	
**Third line treatment**	37 (12%)	16 (6%)	0.353	72 (17%)	16 (4%)	0.149	41 (8%)	19 (4%)	0.342	50 (10%)	16 (3%)	0.491
% of third line treatments received Platinum doublet Single agent Targeted therapy Immunotherapy	3 (8%) 17 (46%) 17 (46%) 0%	0% 6 (38%) 10 (62%) 0%		7 (10%) 32 (44%) 33 (46%) 0%	2 (13%) 8 (50%) 5 (31%) 1 (6%)		4 (10%) 15 (36%) 11 (27%) 11 (27%)	0% 9 (47%) 7 (37%) 3 (16%)		12 (24%) 16 (32%) 11 (22%) 11 (22%)	5 (31%) 2 (13%) 5 (31%) 4 (25%)	

In addition to the change in uptake of treatment, the types of treatment received in first and subsequent lines of therapy changed across the four time cohorts (Table 2). The proportion of patients who received platinum-doublet as first line treatment decreased over time for both age cohorts while more patients received either targeted therapy or ICI upfront. The most notable change occurred between 2015 and 2017 in the second line setting when the receipt of ICI increased from 17% to 53% for patients age <70 years and from 14% to 60% for patients age ≥70 years. The proportion of patients who received platinum-doublet amongst those who received third line therapy also significantly increased in 2017 in both age cohorts (10%–24% for patients age <70 years and 0%–31% for patients age ≥70 years respectively).

Logistic regression was conducted to examine predictors of systemic therapy use. Multivariate analysis (MVA) noted that for both younger and older groups, ECOG PS ≥, smoking history (current or former) and SCS ≥9 were the strongest predictors for decreased use of systemic therapy. Examination of the time cohorts suggested an upward trend of systemic therapy use that was significant for patients age <70 years in 2011 and for patients age ≥70 years in 2011 and 2015 (Table 3).

**Table 3:** Multivariate analysis of systemic therapy uptake in adults age < 70 years and ≥ 70 years

	Age < 70 years		Age ≥ 70 years	
	OR (95% CI)	*p*-value	OR (95% CI)	*p*-value
Sex Male Female	REFERENCE 1.20 (0.97–1.48)	0.094	REFERENCE 0.83 (0.65–1.06)	0.143
**ECOG** **0–1** **≥2** **Unknown**	REFERENCE 0.21 (0.15–0.30) 0.84 (0.60–1.17)	< 0.001 0.292	REFERENCE 0.13 (0.08–0.21) 0.58 (0.356–0.91)	< 0.001 0.017
**SCS** **<9** **≥9**	REFERENCE 0.69 (0.54–0.87)	0.002	REFERENCE 0.80 (0.61–1.05)	0.109
**Year** **2009** **2011** **2015** **2017**	REFERENCE 1.47 (1.08–2.01) 1.02 (0.77–1.36) 1.10 (0.83–1.44)	0.016 0.884 0.520	REFERENCE 1.67 (1.15–2.42) 1.44 (1.03-2.00) 1.17 (0.87–1.57)	0.007 0.033 0.372
**Smoking** **Never** **Current/Former** **Unknown**	REFERENCE 0.25 (0.10–0.63) 0.77 (0.31–1.92)	0.004 0.579	REFERENCE 0.19 (0.08–0.49) 0.46 (0.19–1.14)	< 0.001 0.093

*OR = Odds Ratio; CI = Confidence Interval; ECOG = Eastern Cooperative Oncology Group; SCS = Simplified Comorbidity Score*

### Overall survival

**Fig. 2:**
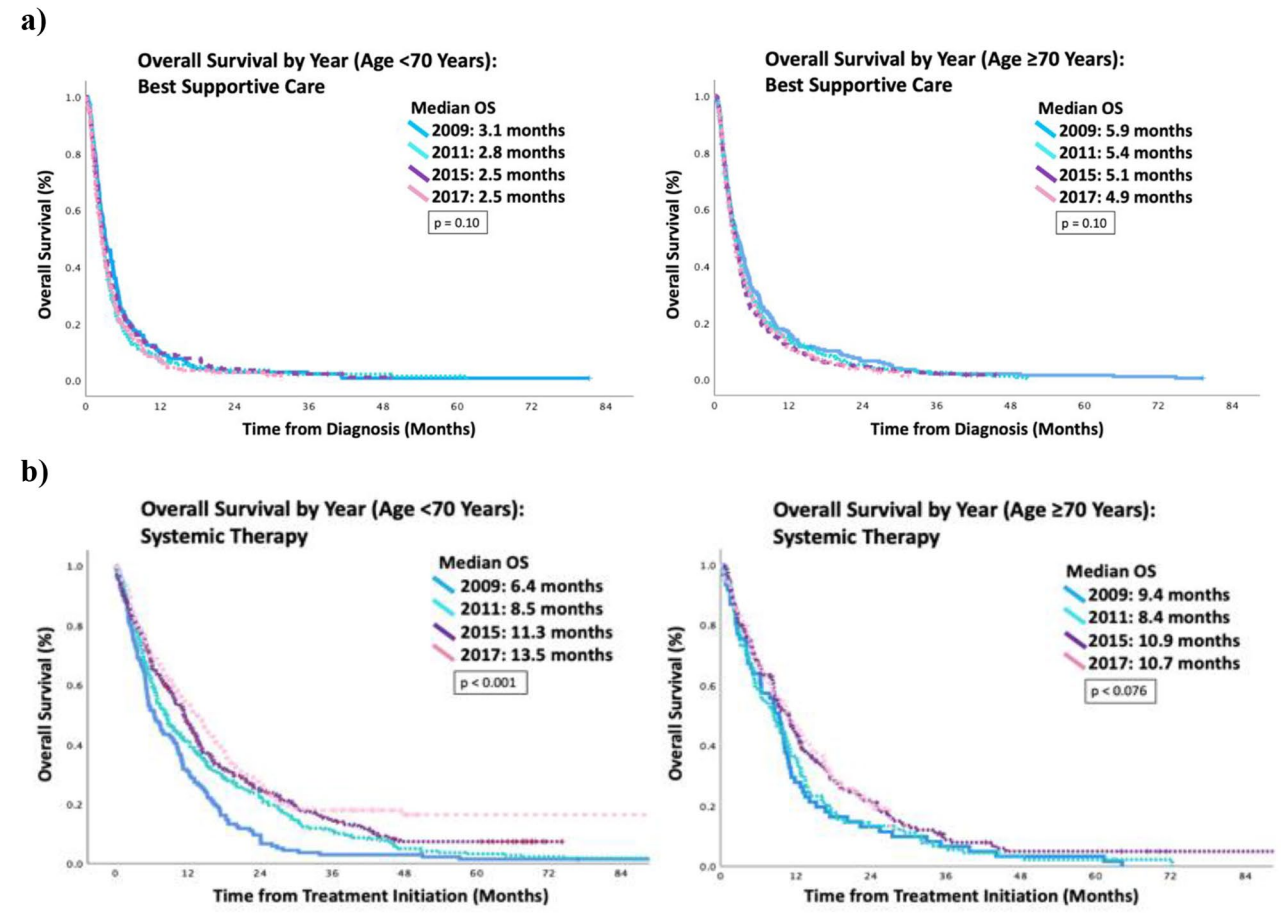
Median overall survival for <70 years and ≥70 years **a**) best supportive care **b**) from initiation of systemic therapy

**Fig. 3:**
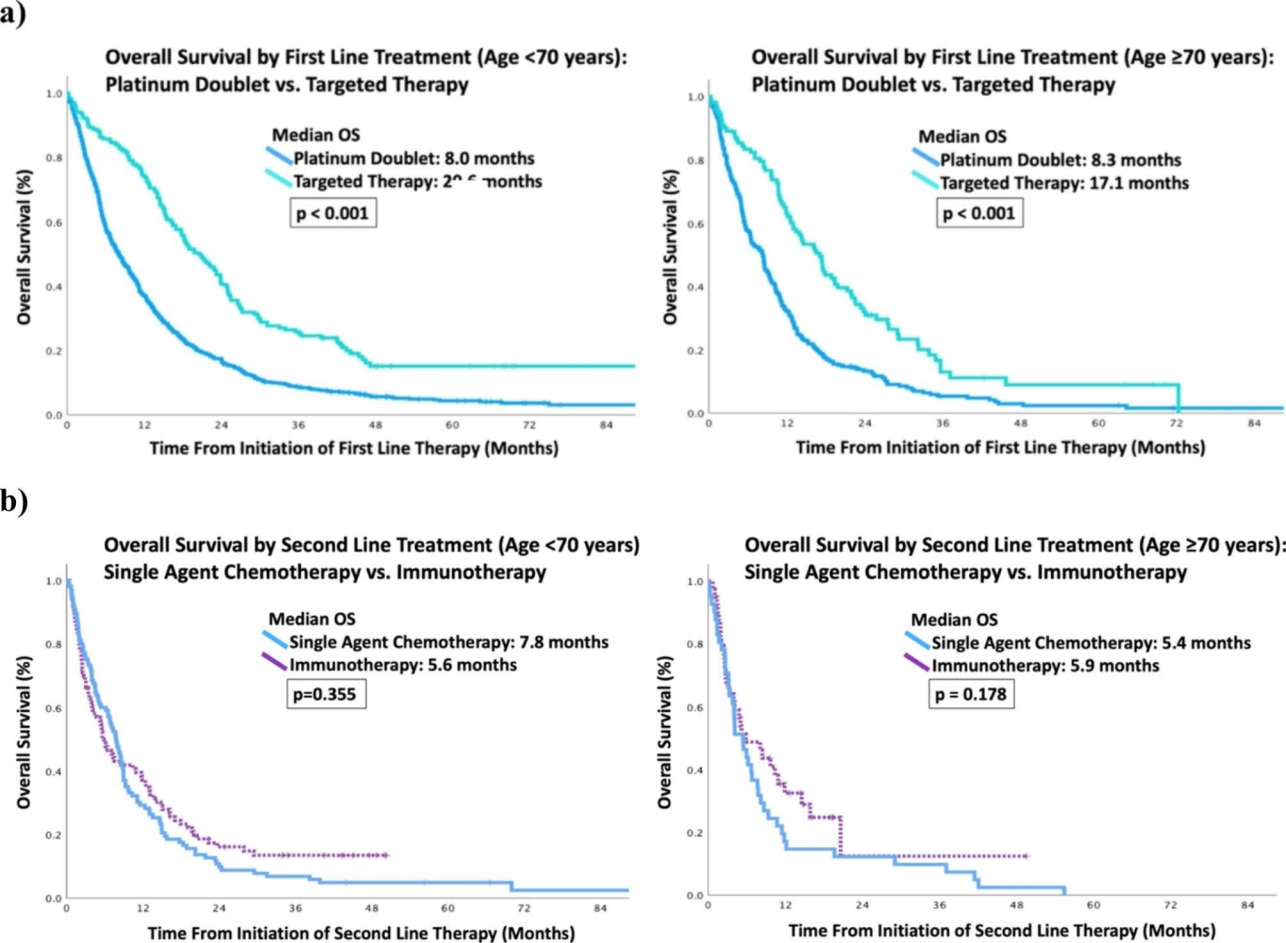
Median overall survival for <70 years and ≥70 years by line of treatment **a**) first line treatment (platinum doublet vs. targeted therapy) **b**) second line treatment (single agent chemotherapy vs. immunotherapy)

Median OS (mOS) for patients who received best supportive care over the four one-year time cohorts was not statistically significantly different. The younger age group had statistically significant improvement in mOS over time for patients who received systemic therapy 6.4 months in 2009, 8.5 months in 2011, 11.3 months in 2015 and 13.5 months in 2017 (*p* < 0.001) but for the older age cohort, the improvement was less robust with 9.4 months in 2009, 8.4 months in 2011, 10.9 months in 2015 and 10.7 months in 2017 (*p* < 0.076). (Fig. 2). The MVA hazard ratio (HR) for OS from diagnosis (Table 4) when controlling for other potential factors signaled an improvement over time compared to baseline but only the 2015 cohort was statistically significant for patients age <70 years (HR 0.82, 95% CI 0.71–0.95, p = 0.008). For the older cohort, there was no difference in 2011 but a positive trend in the subsequent years; neither were statistically significant. We further analyzed mOS starting from the time of initiation of first- and second-line therapy (Fig. 3). In the first line setting, both age cohorts had improved mOS for patients who received targeted therapy compared to platinum doublet: 20.6 months vs. 8 months for patients age <70 years (p < 0.001) and 17.1 months vs. 8.3 months for patients age ≥70 years (p < 0.001). In the second line setting, there was no statistical difference between single agent chemotherapy and ICI in both age groups: ICI 5.6 months vs. 7.8 months for patients <70 years (p = 0.355) and 5.9 months vs. 5.4 months for patients ≥70 years (p = 0.178).

**Table 4:** Multivariate Analyses of overall survival for patients with advanced non-small cell lung cancer: age < 70 years versus ≥70 years

	Age <70 years (n = 1691)		Age ≥70 years (n = 1634)	
	HR (95% CI)	*p*-value	HR (95% CI)	*p*-value
Diagnosis Age 2009 2011 2015 2017	Reference 0.89 (0.76–1.03) 0.82 (0.71–0.95) 0.88 (0.75–1.02)	0.121 0.008 0.085	Reference 1.00 (0.86–1.18) 0.97 (0.84–1.13) 0.87 (0.75–1.02)	0.958 0.714 0.082
**Sex** Male versus Female	1.28 (1.16–1.42)	< 0.001	1.15 (1.04–1.28)	0.008
**ECOG at Diagnosis** PS 0–1 PS ≥2 Unknown	Reference 2.00 (1.79–2.23) 2.13 (1.80–2.51)	< 0.001	Reference 2.14 (1.89–2.41) 1.86 (1.56–2.22)	< 0.001
**SCS** ≥9 versus < 9	1.05 (0.94–1.18)	0.364	0.96 (0.86–1.08)	0.964
**Smoking History** Never Current/Former Unknown	Reference 1.55 (1.34–1.80) 1.72 (1.09–2.70)	< 0.001 0.019	Reference 1.53 (1.30–1.81) 1.66 (1.21–2.28)	< 0.001 0.002

* h = Hazard Ratio; CI = Confidence Interval; ECOG = Eastern Cooperative Oncology Group; PS = Performance Status; SCS = Simplified Comorbidity Score*

## Discussion

In this real-world retrospective cohort study of patients with stage IV NSCLC, the introduction of molecular testing, targeted therapy and immunotherapy were associated with an increased uptake of these therapies regardless of age. Use of systemic therapy was tempered by ECOG PS and smoking history in both younger and older adults, and SCS only in the younger cohort. In our study, the use of systemic treatment resulted in improved survival gains in the <70 years cohort that were mirrored by the ≥70 years group. Our study suggests that in the real world, appropriate patients ≥70 years should be offered systemic therapy with the expectation of similar improvements in OS compared to their younger counterparts.

While lung cancer is more prevalent in the older adult population, in the earlier time cohorts of our study, fewer patients in the older group were seen compared to their younger counterpart (2009: 269 versus 311 and 2011: 361 versus 417). This may reflect a lower likelihood for referral to medical oncology due to ageism, either by the patient or health care providers [20], and the perceived futility of treatment for advanced lung cancer at the time of limited options. In the subsequent two time-cohorts, there was an increase in the number of older patients seen (2015: 516 versus 485 and 2017: 488 versus 478), which may signify a shift in the perception of lung cancer treatment options, particularly for the older adult population.

In our study, approximately half of the younger cohort and the majority of the older cohort received best supportive care only. This is consistent with other population-based studies that have examined the uptake of systemic therapy for advanced NSCLC [21, 22]. For patients who received treatment, the pattern of systemic therapy uptake differed between the two age groups. There was an increasing uptake of systemic therapy for adults younger than 70 years of age with the introduction of EGFR and ALK TKIs, and ICI, whereas this uptake of ALK TKIs was less robust in the 70 years and older group. This may reflect the younger age of patients who are more likely to harbour ALK rearrangement [9, 16, 17, 23, 24]. While the proportion of patients who received second- and third-line therapy increased in 2011, in the subsequent years, the proportions were lower in both age cohorts. This may reflect improved outcomes with first line therapy. For patients who received second line therapy, the proportion of patients decreased in 2015 but mildly increased again in 2017 for both age cohorts. This may be related to the increased availability of ICI in the second line setting in 2017.

The uptake of treatment was influenced by the significant proportion of patients with poor ECOG PS and smoking history. In the younger group, this was compounded by SCS ≥9, indicating these patients had more comorbidities which may further limit the use of systemic therapy. The multivariate analysis suggested these factors were the strongest predictors of the non-use of systemic treatment; more influential than the availability of newer treatment agents. While poor ECOG PS and multimorbidity are associated with increased vulnerabilities to stressors, such as cancer symptoms and cancer treatment-related toxicities, they alone, are insufficient to determine suitability for systemic therapy.

The older adult population is very heterogeneous and have complex and unique care needs. The comprehensive geriatric assessment is a multi-dimensional, interdisciplinary diagnostic process to identify the care needs of older patients that are often undetected with standard clinical assessment [25, 26]. By identifying geriatric syndromes, clinicians can intervene with supportive care and tailor cancer treatment decisions to the patient’s ability to tolerate treatment, which in turn, improves outcomes, such as reducing complications/toxicity, health resource utilization, and mortality [27]. Certain subgroups of older patients may be under- or over-treated when clinical decisions are made solely based on ECOG PS and patients’ comorbidities [28].

Although there was a smaller proportion of older adults who received systemic therapy, those who received treatment had comparable OS to their younger counterpart. The median OS for patients who received best supportive care over the four one-year time cohorts was consistent over time. This data help to ensure that the populations are comparable over time and not unduly influenced by other factors such as stage migration, implementation of lung cancer screening, and other diagnostic technological advances. Interestingly, the median OS for best supportive care in the younger cohort was shorter than the older cohort, raising the possibility of disease aggressiveness in younger adults or disease indolence in older patients. Encouragingly, OS improved over time in both age groups with access to newer therapies, particularly with the introduction of targeted therapy. This suggests that regardless of age, patients appropriately selected can benefit from systemic treatment.

The limitations of this study include the retrospective nature, referral rates to medical oncology, and lack of insight into psychosocial barriers to treatment. The AJCC Lung Cancer Staging System changed over from the Cancer 6th edition to the 7th edition in 2009, which reclassified patients with pleural or pericardial nodules/effusions from stage III to stage IV [29, 30]. The improvement in OS observed in the subsequent time cohorts could potentially be a result of the Will Rogers Phenomenon. Due to the nature of the study design and the potential lag in implementation of new therapies, the changes in outcomes may not be reflective of the changes in access to novel systemic therapy agents that became available during the year when data was collected. A significant confounder was that nivolumab and pembrolizumab were funded after platinum-based therapy, introducing a selection bias for those well enough to receive second line treatment. Our study strengths include examination of a population-based cohort in a centralized, single payer health care system governed by provincial guidelines for cancer therapies. There were no financial barriers in accessing cancer treatments given the universal health care system in Canada, providing a realistic assessment of treatment rates of patients with advanced NSCLC.

## Conclusion

Our study demonstrated the benefits of systemic therapy were seen across both age groups with improvements associated with the introduction of newer therapeutic options. This suggests that, with careful assessment and selection of appropriate candidates, older adults with advanced NSCLC should receive equitable access to systemic therapy. In future studies, patient-reported outcomes relevant to the older adult population should be included, such as maintenance of independence, quality of life and function.

## Data Availability

The datasets used and analyzed during the current study are available from the corresponding author upon reasonable request.

## Abbreviations

ALK: Anaplastic lymphoma kinase
B.C.: British Columbia
ECOG: Eastern Cooperative Oncology Group
EGFR: Epidermal growth factor receptor
ICI: Immune checkpoint inhibitors
NSCLC: Non-small cell lung cancer
OASIS: Outcomes and Surveillance Integrated System
OS: Overall survival
PD-1: Programmed death – 1
PD-L1: Programmed death-ligand – 1
PS: Performance status
SCS: Simplified comorbidity score
ST: Systemic therapy
TKI: Tyrosine kinase inhibitor
